# Anaemia and all-cause mortality in contemporary heart failure with preserved ejection fraction (left ventricular ejection fraction ≥50%): an updated systematic review and meta-analysis

**DOI:** 10.1093/eschf/xvag213

**Published:** 2026-09-08

**Authors:** Suyash Tripathi, Dharmendra Jain

**Affiliations:** Department of Cardiology, Institute of Medical Sciences, Banaras Hindu University, Varanasi 221005, Uttar Pradesh, India; Department of Cardiology, Institute of Medical Sciences, Banaras Hindu University, Varanasi 221005, Uttar Pradesh, India

**Keywords:** Heart failure with preserved ejection fraction, Anaemia, Haemoglobin, Mortality, Meta-analysis, Prognosis

## Abstract

**Aims:**

Anaemia is among the most prevalent comorbidities in heart failure with preserved ejection fraction (HFpEF), but pooled prognostic estimates predate the contemporary left ventricular ejection fraction (LVEF) ≥50% definition and combine legacy with contemporary cohorts. We quantified the association between World Health Organization (WHO)-defined anaemia and all-cause mortality in HFpEF and tested whether it persists under the stricter ejection-fraction threshold.

**Methods and results:**

Following PRISMA 2020 (PROSPERO CRD420251361160), we searched five databases from inception to 2026 and included cohort studies reporting an adjusted hazard ratio (aHR) for binary WHO-defined anaemia vs all-cause mortality in an ejection fraction-stratified HFpEF population; every estimate was verified against its primary source and screening was duplicated (*κ* = 1.00 after adjudication). Of 6123 records, 20 studies were included; 17 (109 909 patients; anaemia prevalence 26%–70%; follow-up 12–72 months) contributed to two pre-specified co-primary pools analysed with Paule–Mandel random-effects models and Hartung–Knapp–Sidik–Jonkman variance correction: a legacy pool (LVEF ≥40%, *k* = 17) and a contemporary pool (LVEF ≥50%, *k* = 12). Anaemia was associated with higher all-cause mortality in both: aHR 1.49 (95% CI 1.35–1.66; 95% prediction interval (PI) 1.07–2.09; *I*^2^ = 85%) in the ≥40% pool and 1.41 (1.26–1.57; PI 1.06–1.87; *I*^2^ = 83%) in the ≥50% pool, both PIs excluding the null. The estimate was stable across 12 pre-specified sensitivity analyses (range 1.45–1.52) with no influential study. Across four Bayesian priors, the posterior median aHR was 1.39–1.49 with posterior probability of harm ≥.999, and a robust Bayesian model-average that entertains null, homogeneity, and publication-bias models returned aHR 1.41 (95% credible interval 1.17–1.58). Although small-study effects were detected (Egger *P* = .03), the bias-corrected estimate was essentially unchanged (PEESE aHR 1.39). A per-1 g/dL lower-haemoglobin dose–response meta-analysis (*k* = 6) yielded aHR 1.17 (1.08–1.27). The accrued sample exceeded the diversity-adjusted required information size, and GRADE certainty was moderate under the prognostic-factor framework.

**Conclusions:**

WHO-defined anaemia is independently associated with a ∼40–50% higher all-cause mortality in HFpEF, preserved under the contemporary LVEF ≥50% definition and robust to extensive sensitivity, Bayesian, and bias-correction analyses. These findings support incorporating anaemia into HFpEF risk assessment and motivate mortality-powered trials in rigorously defined HFpEF, while not implying that anaemia correction improves outcomes.

**Registration:**

PROSPERO CRD420251361160.

## Graphical Abstract

**Graphical Abstract:**
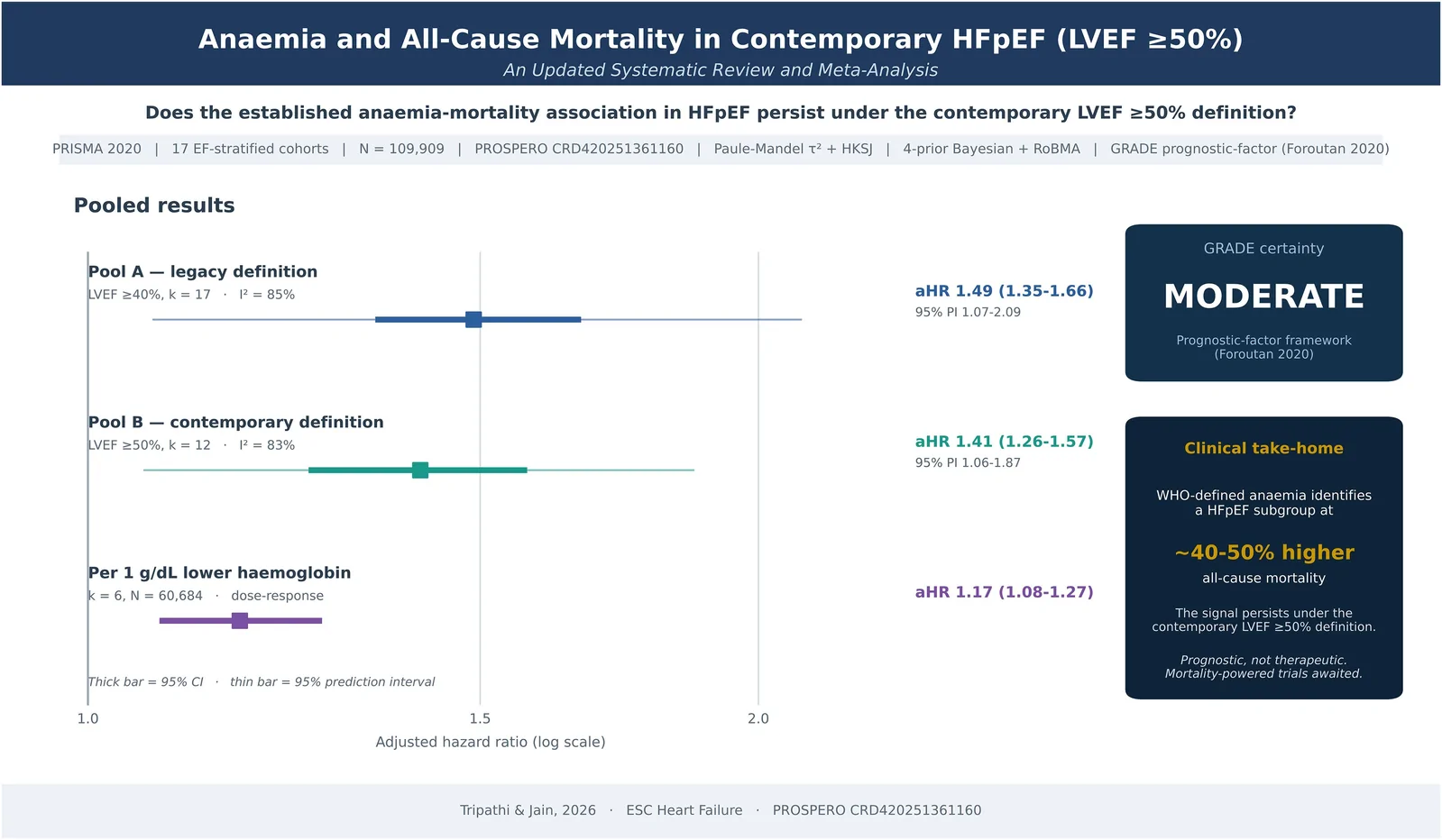

## Introduction

Heart failure with preserved ejection fraction (HFpEF) now accounts for at least half of all heart failure and is increasingly defined, in current European and American guidance, by a left ventricular ejection fraction (LVEF) ≥ 50%, a stricter threshold than the ≥40%–45% cut-offs used in much of the foundational literature.^1^ HFpEF is conceived as a systemic, comorbidity-driven syndrome in which extracardiac conditions provoke microvascular inflammation and myocardial dysfunction.^2^ Anaemia is among the most prevalent of these comorbidities, affecting a third to two-thirds of patients across cohorts, and is biologically plausible as a mortality driver through reduced oxygen delivery, neurohormonal and renal activation, and elevated cardiac workload.

Individual cohorts and an individual-patient-data analysis from the MAGGIC consortium have linked anaemia to worse outcomes across the ejection-fraction spectrum,^3^ and a 2021 aggregate meta-analysis reported an association in HFpEF.^4^ However, the available syntheses pre-date the LVEF ≥50% consensus and pool legacy ≥40% cohorts with contemporary ones. Whether the prognostic signal survives under the contemporary definition, and how robust it is to statistical assumptions and small-study effects, remains unresolved. This question is clinically timely because intravenous iron therapy reduces hospitalization in iron-deficient heart failure,^5^ sharpening the need to know whether anaemia itself marks excess mortality risk in rigorously defined HFpEF.

We therefore performed an updated systematic review and meta-analysis with two pre-specified co-primary analyses: (i) a legacy LVEF ≥40% pool and (ii) a contemporary LVEF ≥50% pool, and triangulated the frequentist result with Bayesian inference, formal publication-bias diagnostics, a dose–response synthesis, and GRADE certainty rating.

## Methods

The review was registered (PROSPERO CRD420251361160) and is reported per PRISMA 2020^6^; the completed checklist, full search strategy, and a deviation log appear in the Supplementary material (§S1.1–§S1.2). We searched PubMed/MEDLINE, Embase, Web of Science, Scopus, and Cochrane CENTRAL from inception to 2026 without language restriction. Records were screened in duplicate (Rayyan); Cohen’s *κ* was .56 before and 1.00 after adjudication.

Eligible studies were cohorts of adults with ejection fraction (EF)-stratified HFpEF. The exposure was binary World Health Organization (WHO)-defined anaemia (Hb <13 g/dL men, <12 g/dL women); the comparator was non-anaemia; the outcome was all-cause mortality; and the eligible effect measure was an adjusted hazard ratio (aHR), or an estimate validly convertible to one. Logistic-regression odds ratios for this common outcome, which cannot be converted to a hazard ratio, were ineligible (§S1.3). Every included estimate was verified against its primary report for model type, effect measure, point estimate, confidence interval, exposure definition, outcome, and EF stratum (§S1.4); estimates available only from a prior meta-analysis were not accepted. Risk of bias was assessed with ROBINS-E across seven domains.

Two co-primary pools were pre-specified: Pool A, the legacy definition (LVEF ≥40%), and Pool B, the contemporary definition (LVEF ≥50%). Both were synthesized with random-effects models using the Paule–Mandel *τ*^2^ estimator and Hartung–Knapp–Sidik–Jonkman (HKSJ) variance correction^7,8^; for each pool, we report the pooled aHR with 95% confidence interval, Cochran’s *Q*, *τ*^2^ and *τ*, *I*^2^, and the 95% prediction interval (PI) (Hartung–Knapp *t*-distribution on the HKSJ-corrected variance). All pools and analyses are reported as estimation endpoints with point estimates and 95% intervals, so no alpha split or family-wise correction was pre-specified. Twelve pre-specified sensitivity analyses (SA-1 to SA-12; full detail in §S1.5) probed estimator choice, definition strictness, influence and outliers, study composition, and anchor transparency, together with small-study and selection-bias diagnostics using a robust Bayesian model-average (RoBMA^9^), a Vevea–Hedges selection model, PEESE-RE/FAT-PET regression,^10^ and a test for excess significance (TES).^11^

Bayesian random-effects meta-analysis used bayesmeta^12^ under four pre-specified priors (weakly informative, Majmundar 2021-anchored, Berry MAGGIC-anchored, and sceptical; full specification in §S1.5), together with an RoBMA.^9^ A cumulative meta-analysis^13^ characterized temporal stabilization, and a secondary per-1 g/dL-lower-haemoglobin dose–response pool was synthesized. Evidence sufficiency was assessed against the diversity-adjusted required information size (DIS),^14^ and certainty was rated with the GRADE framework for prognostic factors, which begins at high certainty for cohort evidence.^15^ Analyses used R 4.6.0 (metafor).

## Results

### Study selection

Of 6123 records identified, 2711 duplicates were removed and 3412 were screened, of which 3327 were excluded at title/abstract. Eighty-five reports underwent full-text assessment; 65 were excluded. Twenty studies entered the review: 17 contributed binary-anaemia aHRs to the co-primary synthesis, and three further cohorts (Okuno 2019,^16^ Tada 2008,^17^ and Young 2008^18^) contributed only to the per-haemoglobin dose–response pool (*Figure 1*). One full-text report (Köseoğlu 2024^19^) was excluded under our pre-specified effect-measure criterion: it reported a multivariable logistic-regression odds ratio (5.40) for a common (28%) outcome, which is not convertible to a hazard ratio; pooling it with the aHRs would have mixed incompatible estimands. Because the excluded estimate was large, the exclusion is conservative; its inclusion would have increased, not reduced, the pooled association. The exclusion and its full rationale are documented in the Supplementary exclusion table (Supplementary Table S1, §S1.8), and it does not enter the synthesis.

**Figure 1:**
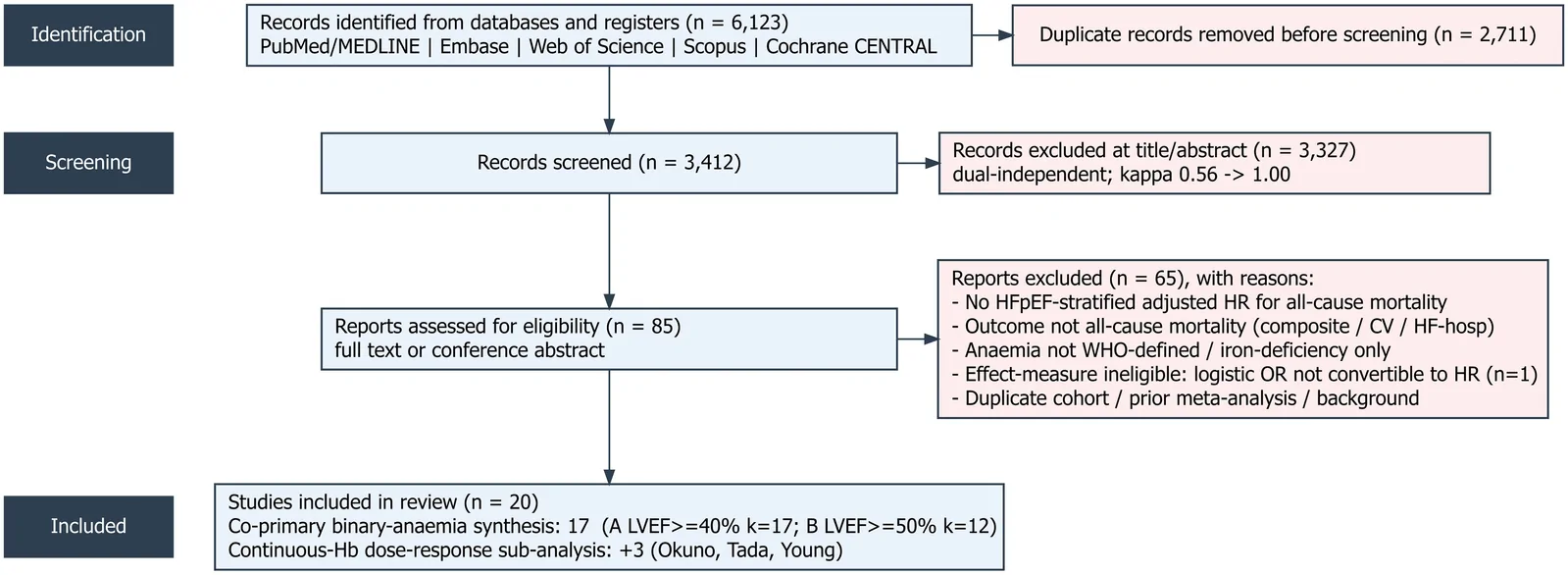
PRISMA 2020 flow diagram.

### Study characteristics and risk of bias

The 17 co-primary studies (*Table 1*) comprised 109 909 patients with HFpEF, spanning 2005–25, with mean ages 64–81 years, anaemia prevalence 26%–70%, and follow-up 12–72 months. Twelve cohorts used the contemporary LVEF ≥50% threshold (Pool B); five legacy cohorts used ≥40%–45% (Pool A only). Anaemia was WHO-defined in 16 cohorts and ICD-9-coded in one (Veterans-HFpEF). On ROBINS-E, 6 cohorts were rated low and 11 moderate risk of bias; the dominant concerns were residual confounding (one cohort adjusted for age and sex only) and one graphically extracted estimate (Supplementary Figure S1).

**Table 1:** Characteristics of the 17 studies in the co-primary synthesis

#	Study	EF cut (%)	N HFpEF	Mean age	% male	An prev (%)	Follow-up (mo)	aHR (95% CI)	Anaemia def	RoB	Pool
1	Grigorian Shamagian 2005^20^	≥50	210	75	48.1	46	12	2.65 (1.31–5.36)	WHO	Mod	A + B
2	Felker 2006^21^	≥40	3093	64	44.5	38	42	1.61 (1.39–1.88)	WHO	Low	A
3	Tehrani 2009^22^	≥50	294	78.2	38	55	40	1.57 (1.09–2.26)	WHO	Mod	A + B
4	Ather 2012^23^	≥50	2843	70.7	91.1	33.2	24	1.35 (1.13–1.61)	WHO	Mod	A + B
5	Caughey 2014^24^	≥40	6414	76	38.8	70	12	2.10 (1.60–2.70)	WHO	Low	A
6	Lund 2014^25^	≥45	539	79	44	51	24.5	1.86 (1.33–2.62)	WHO	Mod	A
7	PEOPLE 2016^26^	≥50	280	73.7	55	46	24	1.33 (0.85–2.09)	WHO	Mod	A + B
8	Veterans-HFpEF 2017^27^	>50	29 022	71	96	35	43.2	1.52 (1.47–1.57)	ICD-9	Mod	A + B
9	Streng 2018^28^	≥50	556	78	54	45.5	25	1.19 (0.85–1.66)	WHO	Mod	A + B
10	Iorio 2018^29^	≥50	1373	79	49	26	31	1.90 (1.40–2.60)	WHO	Mod	A + B
11	Jin 2019^30^	≥50	1604	74.3	53.1	51	33.9	1.14 (0.85–1.52)	WHO	Low	A + B
12	Gupta 2020^31^	≥45	1748	72	∼50	41	28.8	1.40 (1.14–1.74)	WHO	Low	A
13	Savarese 2020^32^	≥50	9956	77.2	45.3	41	26.4	1.28 (1.20–1.36)	WHO	Low	A + B
14	Chairat 2020^33^	≥50	287	64	40	67.6	12	2.67 (1.22–5.85)	WHO	Mod	A + B
15	Pintér 2022^34^	≥40	375	75	48	37	16.8	2.33 (1.21–4.52)	WHO	Mod	A
16	Qiu 2023^35^	≥50	48 992	72.2	50	48.1	12	1.30 (1.21–1.38)	WHO	Low	A + B
17	Ugarph-Morawski 2025^36^	≥50	2323	81	55	28.8	72	1.41 (1.27–1.57)	WHO	Mod	A + B

Pool A (LVEF ≥40%) = all 17; Pool B (LVEF ≥50%) = the 12 rows marked A + B. aHR, adjusted hazard ratio; An prev, anaemia prevalence; RoB, ROBINS-E overall.

### Co-primary meta-analyses

In the legacy pool (Pool A, LVEF ≥40%, *k* = 17), anaemia was associated with higher all-cause mortality: aHR **1.49 (95% CI 1.35–1.66)**, with substantial heterogeneity (Cochran's *Q* = 60.62, df = 16, *P* < .001; *I*^2^ = 85%; *τ*^2^ = .022, *τ* = .150) and a 95% PI (Hartung–Knapp) of 1.07–2.09 that excluded the null (*Figure 2A*). In the contemporary pool (Pool B, LVEF ≥50%, *k* = 12), the association was preserved and only marginally attenuated: aHR **1.41 (1.26–1.57)**, Cochran's *Q* = 46.20, df = 11, *P* < .001; *I*^2^ = 83%; *τ*^2^ = .015, *τ* = .121; PI 1.06–1.87 (*Figure 2B*). The two largest cohorts (Qiu 2023^35^ and Veterans-HFpEF 2017^27^) and Savarese 2020^32^ carried the most weight (≈10% each), but no single study dominated (Supplementary Table S2).

**Figure 2:**
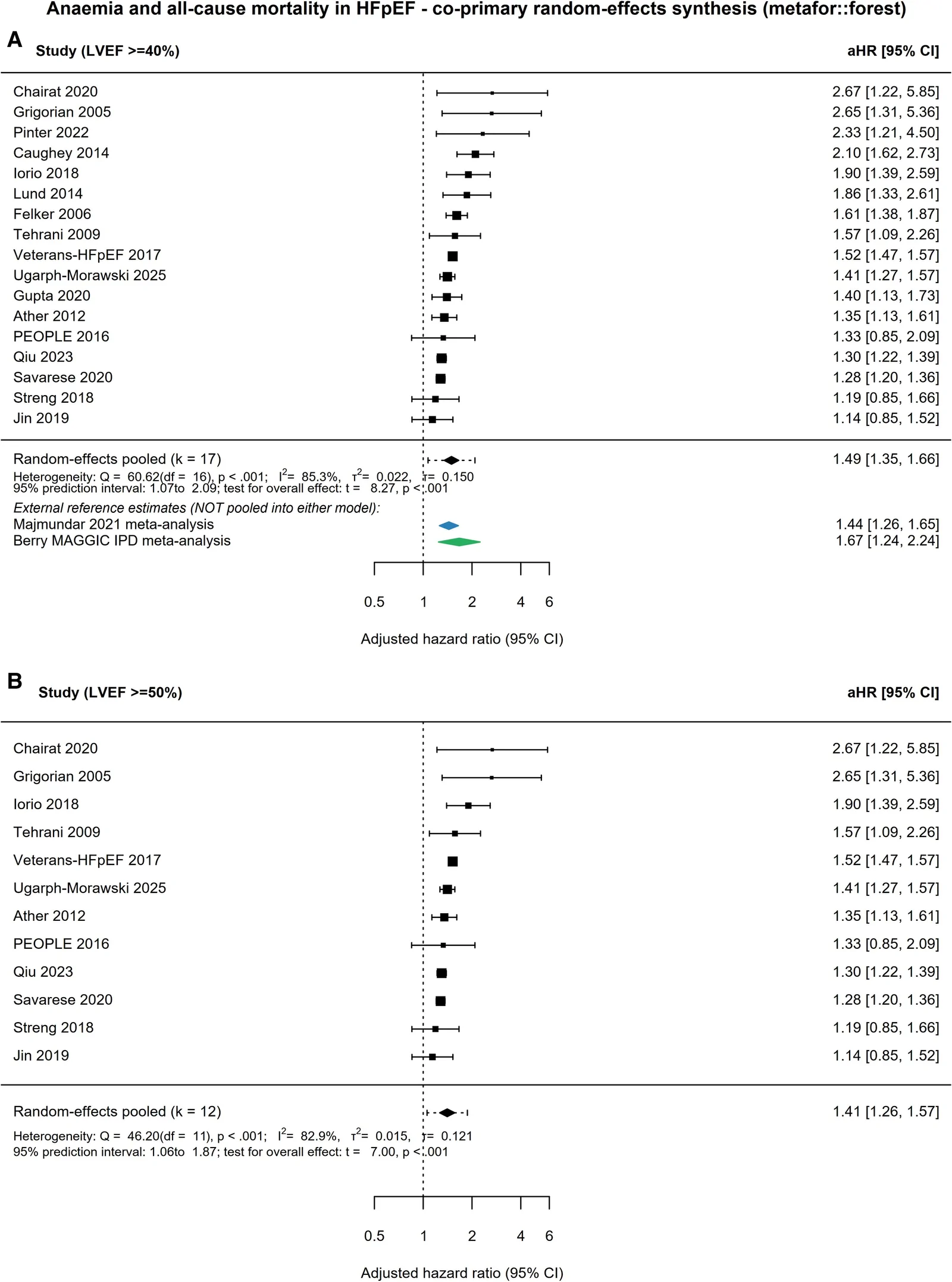
Co-primary random-effects forest plots with study-level adjusted hazard ratios and 95% confidence intervals (HR [95% CI] column at right): panel A (LVEF ≥40%, *k* = 17) and panel B (LVEF ≥50%, *k* = 12). Black squares are study estimates (area ∝ weight); the black diamond is the random-effects pooled estimate. In panel A, two external benchmark estimates are shown for reference only and are **not pooled** into either model: the Majmundar 2021 aggregate meta-analysis (HR 1.44 [1.26–1.65]) and the Berry MAGGIC individual-patient-data meta-analysis (HR 1.67 [1.24–2.24]); both are labelled directly on the plot. Heterogeneity statistics (*Q*, df, *P*, *I*^2^, *τ*^2^, *τ*), the 95% prediction interval (dashed line on the diamond), and the test for overall effect are annotated beneath each pooled estimate.

### Sensitivity analyses

The pooled estimate was stable across the pre-specified sensitivity registry (SA-1 to SA-12; *Table 2*). Alternative *τ*^2^ estimators gave 1.47 (DerSimonian–Laird) and 1.48 (REML); restricting to WHO-measured anaemia (dropping the single ICD-coded cohort) gave 1.50 (1.33–1.68); excluding conference-abstract sources gave 1.52 (1.35–1.72); restricting to cohorts with ≥500 HFpEF patients gave 1.45 (1.31–1.62); and adding the Berry MAGGIC individual-patient-data estimate as a transparency entry gave 1.50 (1.36–1.65). A pre-specified outlier/influence diagnostic (studentized deleted residuals with Bonferroni correction, Cook's distance, Baujat plot, and leave-one-out) identified no statistical outlier, the largest residual (Caughey 2014,^24^ |*z*| = 1.99) did not reach the corrected threshold (2.97) and no cohort was influential, so the conditional outlier-excluded re-pool (SA-5) was not performed; leave-one-out estimates ranged narrowly from 1.45 to 1.52 (Supplementary Table S4, Supplementary Figure S2). Every re-pooling analysis kept the point estimate within 1.45–1.52 with confidence and PIs excluding the null; the publication-bias suite (SA-9 to SA-12) is reported in the ‘Publication bias and small-study effects’ section. A cumulative meta-analysis showed the estimate stabilizing near 1.5 from 2014 onward (Supplementary Figure S3).

**Table 2:** Sensitivity analyses (Pool A unless stated)

Code	Analysis	*k*	aHR (95% CI)	95% PI	*I* ^2^ (%)
Primary-A	Co-primary A (LVEF ≥40%), PM *τ*^2^ + HKSJ	17	1.49 (1.35–1.66)	1.07–2.09	85
Primary-B	Co-primary B (LVEF ≥50%), PM *τ*^2^ + HKSJ	12	1.41 (1.26–1.57)	1.06–1.87	83
SA-1	DerSimonian–Laird *τ*^2^ + HKSJ	17	1.47 (1.34–1.61)	1.16–1.87	74
SA-2	REML *τ*^2^ + HKSJ	17	1.48 (1.34–1.63)	1.13–1.93	78
SA-3	WHO-strict: drop ICD-coded anaemia (Veterans-HFpEF/Imran)	16	1.50 (1.33–1.68)	1.04–2.15	79
SA-4	Outlier/influence diagnostic (studentized residuals, Cook's *D*, Baujat, LOO)	17	No outlier (max|*z*| = 1.99, Caughey 2014; threshold 2.97); LOO 1.45–1.52		
SA-5	Outlier-excluded re-pool (conditional on SA-4)		Not done—no statistical outlier		
SA-6	Exclude conference-abstract sources (Imran, Qiu)	15	1.52 (1.35–1.72)	1.05–2.21	73
SA-7	Restrict to N HFpEF ≥500	12	1.45 (1.31–1.62)	1.05–2.01	88
SA-8	Add Berry MAGGIC IPD transparency entry	18	1.50 (1.36–1.65)	1.09–2.05	83
SA-9	Robust Bayesian Meta-Analysis (model-averaged)	17	1.41 (1.17–1.58) CrI		
SA-10	Vevea–Hedges 4-parameter selection model	17	1.42 (adj)		
SA-11	PEESE-RE (Stanley–Doucouliagos)	17	1.39 (adj)		
SA-12	Test for Excess Significance	17	O/E 1.15, *P* = .20		

SA codes follow the pre-specified protocol (SA-1…SA-12), applied to Pool A unless stated.

PI, 95% prediction interval; CrI, credible interval; adj, bias-adjusted point estimate. **SA-4** is the outlier/influence diagnostic: no study exceeded the Bonferroni-corrected studentized-residual threshold (|*z*| > 2.97), and no case was influential (Cook's distance), so **SA-5** (the outlier-excluded re-pool, which is conditional on SA-4) was not triggered. **SA-9 to SA-12** are the publication-bias suite, detailed in Supplementary Tables S6–S9; for SA-9, the inclusion Bayes factor for a non-null effect was 79. Every re-pooling analysis (SA-1, 2, 3, 6, 7, 8) kept the point estimate within 1.45–1.52 with confidence and prediction intervals excluding the null; bias-adjusted estimates ranged 1.39–1.42.

### Bayesian inference

Bayesian random-effects models concurred: across the four pre-specified priors (§S1.5) the posterior median aHR was 1.47–1.49 in Pool A and 1.39–1.42 in Pool B, with posterior probability of harm (HR >1) ≥ .999 in every case (*Figure 3*, Supplementary Table S5); the externally anchored priors moved the estimate by ≤.02 and the sceptical prior did not pull it towards the null. Because the Majmundar 2021 meta-analysis shares nine of our Pool-A cohorts (and incorporates the Berry MAGGIC estimate), these informative-prior analyses are reported as robustness-to-anchoring checks, not confirmatory evidence (see the ‘Strengths and limitations’ section, §S1.5). A robust Bayesian model-average that entertains null, homogeneity, and publication-bias models returned a model-averaged aHR of 1.41 [95% credible interval (CrI) 1.17–1.58] (Supplementary Table S6, Supplementary Figure S4), confirming the association survives when the analysis is allowed to shrink it towards the null.

**Figure 3:**
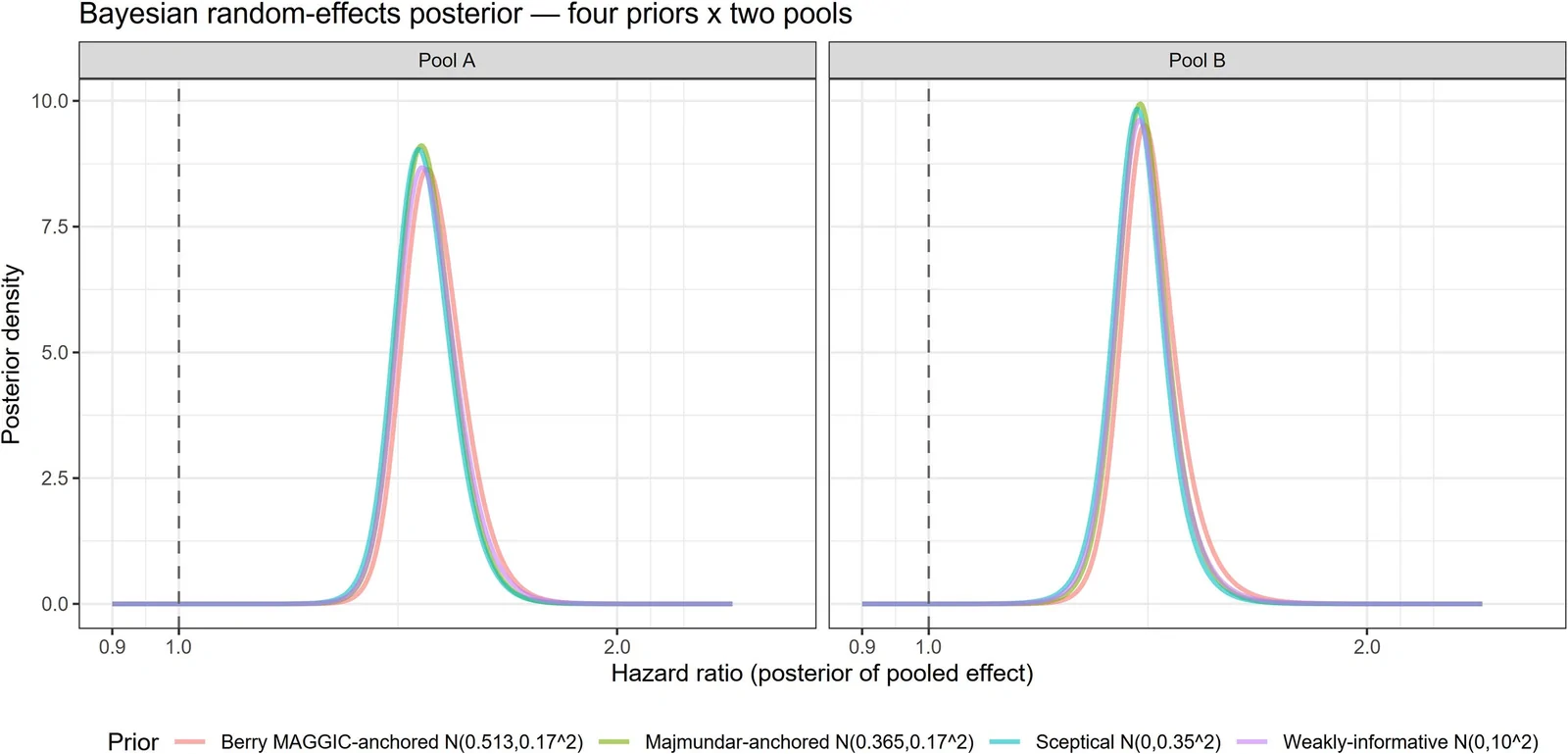
Bayesian random-effects posterior densities of the pooled hazard ratio under the four pre-specified priors (weakly informative, Majmundar-anchored, Berry MAGGIC-anchored, sceptical), by pool.

### Publication bias and small-study effects

A FAT-PET regression (Stanley–Doucouliagos formulation of Egger's test) indicated modest funnel asymmetry (slope = 1.23, *P* = .031); bias-corrected estimates remained clearly positive (PEESE-RE adjusted aHR 1.39, 95% CI 1.27–1.52; Vevea–Hedges selection model 1.42; Supplementary Tables S7 and S8, Supplementary Figures S5 and S6). The TES was negative (14 observed vs 12.2 expected significant studies; *z* = 1.29, *P* = .20; Supplementary Table S9), arguing against selective-reporting inflation.

### Per-haemoglobin-unit dose–response

A formal per-1 g/dL-lower-haemoglobin pool (*k* = 6: Okuno 2019,^16^ Tada 2008,^17^ and Young 2008^18^ as dose–response-only cohorts, plus Caughey 2014,^24^ Felker 2006,^21^ and Gupta 2020^31^ which also contributed to the binary pool; 60 684 patients) yielded aHR 1.17 (1.08–1.27) for all-cause mortality (Supplementary Table S10, Supplementary Figure S7), demonstrating a graded relationship consistent with the binary-anaemia signal. This dose–response synthesis is based on six cohorts, three of which also contribute to the binary pool and which show moderate heterogeneity (*I*^2^ ≈ 67%); it is interpreted as supportive, consistency evidence rather than independent confirmation.

### Heterogeneity exploration

All six pre-specified moderators (EF threshold, publication year, follow-up duration, mean age, anaemia prevalence, proportion male) were tested univariably; with *k* = 17 studies and six candidate moderators, a multivariable specification would be overfit per Borenstein guidance (*k* ≥ 10 per moderator), and meta-regression results are reported as hypothesis-generating. Univariable meta-regression identified the ejection-fraction threshold as the only moderator approaching significance (*β* = −.20 per ≥50% vs <50% stratum, *P* = .057, *R*^2^ = 36%): permissive <50% cohorts (*k* = 5) pooled higher at 1.71 (1.36–2.14) than strict ≥50% cohorts (*k* = 12) at 1.41 (1.26–1.57) (Supplementary Tables S11 and S12, Supplementary Figure S8). Publication year, follow-up, mean age, anaemia prevalence, and proportion male were not significant moderators. Heterogeneity therefore reflects, in part, the EF definition itself rather than measured clinical covariates.

### Evidence sufficiency and certainty

The accrued sample (109 909) far exceeded the DIS. At the primary anchor scenario (HR 1.49 as observed, baseline non-anaemic 1-year mortality .30, *α* = .05 two-sided, power = .80), DIS = 5181 (*D*^2^ = .85; Supplementary Table S13); across alternative anchors (HR 1.20–1.49, baseline .20–.40), DIS ranged 4010–39 907. The accrued sample therefore exceeds the most conservative DIS scenario by a factor of 2.75. Under the GRADE prognostic-factor framework, which starts at high certainty for cohort evidence, certainty was rated Moderate. We downgraded one level for risk of bias (11 of 17 cohorts moderate on ROBINS-E, with variable adjustment, one cohort adjusted for age and sex only, one ICD-coded anaemia definition, one graphically extracted estimate, and two conference-abstract sources, none high or critical) and one level for inconsistency (*I*^2^ ≈ 85% with substantial between-cohort variation in adjusted point estimates). We did not downgrade for indirectness or imprecision; although small-study effects were detected, the bias-corrected estimates were stable, so this was carried as a limitation rather than a separate downgrade. We applied a one-level upgrade for the graded per-1 g/dL-lower-haemoglobin dose–response relationship (*Table 3*, Supplementary Table S14). A more conservative assessor who does not consider the dose–response sufficient for upgrading would rate the certainty Low.

**Table 3:** GRADE evidence profile (prognostic-factor framework)

Domain	Judgement
Study design	Cohort/registry—starts HIGH (Foroutan 2020)
Risk of bias	Serious (−1)—11/17 cohorts moderate on ROBINS-E; variable adjustment, one ICD-coded definition, one graphically extracted estimate, two conference abstracts; none high
Inconsistency	Serious—*I*^2^ ≈ 85%, downgrade 1 level
Indirectness	Not serious—direct HFpEF, all-cause mortality
Imprecision	Not serious—Pool A 95% CI 1.35–1.66 excludes null
Publication bias	Not serious—Egger modest, PEESE/RoBMA stable, TES negative
Large effect/dose–response	Dose–response upgrade (+1)—graded per-1 g/dL gradient (HR 1.17, 1.08–1.27); large-effect criterion not met (HR <2.0)
**Overall certainty**	Moderate (high −1 risk of bias −1 inconsistency +1 dose–response)—aHR 1.49 (1.35–1.66) Pool A; 1.41 (1.26–1.57) Pool B

## Discussion

### Main findings

Across contemporary and legacy cohorts, WHO-defined anaemia was independently associated with a ∼40%–50% higher all-cause mortality in HFpEF. Prior syntheses had already established this association across the ejection-fraction spectrum; our specific contribution is to quantify it under the contemporary LVEF ≥50% definition and to show how the estimate changes between the legacy ≥40% and contemporary ≥50% thresholds. The central new finding is that this association persists under the contemporary LVEF ≥50% definition (1.41) with only modest attenuation from the legacy ≥40% estimate (1.49). The result was concordant across frequentist, Bayesian, and model-averaged analyses, stable across the pre-specified SA-1 to SA-12 panel (SA-5 not performed since SA-4 identified no statistical outlier) including leave-one-out, supported by a graded per-haemoglobin dose–response, and robust to publication-bias correction.

### Comparison with prior evidence

Our estimate aligns with, but refines, the 2021 aggregate meta-analysis^4^ and the MAGGIC individual-patient analysis.^3^ The separation of legacy and contemporary EF definitions is, to our knowledge, novel and directly addresses the criticism that older pooled estimates may not transport to guideline-defined HFpEF.

### Heterogeneity and interpretation

Statistical heterogeneity was high, but its direction was invariant: every cohort and every PI lay above the null. Meta-regression localized a meaningful share of heterogeneity to the EF threshold, with more permissive (<50%) cohorts showing larger associations, plausibly because they admit patients with heart failure with mildly reduced ejection fraction patients in whom anaemia and the underlying syndrome carry different prognostic weight. This supports reporting the ≥50% estimate as the contemporary reference value, while recognizing that contemporary disease-modifying therapy in HFpEF (sodium-glucose co-transporter 2 inhibition) improves health status broadly across major subgroups including anaemia,^37^ and that future cohorts treated with such therapy may show a smaller absolute mortality gap than the historical cohorts pooled here. This EF-threshold association is hypothesis-generating only: it did not reach statistical significance (*P* = .057), derives from a univariable model that cannot adjust for collinear study-level factors, and should not be read as establishing the ejection-fraction threshold as the cause of the heterogeneity.

### Clinical implications

A conceptual clarification is essential: this synthesis concerns WHO-defined anaemia as a prognostic marker, which is distinct from iron deficiency, the target of current heart-failure therapeutics, and iron deficiency may occur with or without anaemia. Anaemia is a simple, universally measured marker that identifies an HFpEF subgroup at materially higher mortality risk. Whether correcting anaemia alters that risk is not answerable from observational data; the association should be read as prognostic, not causal. Critically, the contemporary HFpEF-targeted evidence on anaemia or iron-deficiency correction is sparse. IRONMAN^5^ and HEART-FID^38^ randomized predominantly heart failure with reduced ejection fraction cohorts (LVEF cut-offs of 45% and 40%, respectively); neither was powered for HFpEF mortality, and the long-term HEART-FID analysis found only a non-significant 18% relative reduction in 12-month all-cause mortality (HR .82, 95% CI .65–1.04).^38^ A dedicated HFpEF iron-deficiency trial is awaited. In Indian and other low- and middle-income settings, the clinical urgency is heightened: a contemporary North-Indian heart-failure cohort reported anaemia in 54% of admissions, yet ferric carboxymaltose was prescribed in only 7.5% at discharge,^39^ a therapeutic gap that our pooled mortality estimate of roughly 40%–50% excess risk renders difficult to ignore. The consistency and magnitude of the mortality signal therefore strengthen the rationale for mortality-powered interventional trials in rigorously defined, contemporary HFpEF; such trials should distinguish anaemia from iron deficiency without anaemia, since the WHO haemoglobin threshold may underestimate true iron-deficient HFpEF in populations with concurrent haemoglobinopathy traits (thalassaemia, sickle trait) that are common in South Asian and African cohorts. What changes for clinicians is modest but concrete: a definition-consistent effect size for risk stratification and trial enrichment, not a treatment recommendation, since no trial has shown that correcting anaemia improves outcomes in HFpEF.

### Strengths and limitations

Strengths include dual co-primary EF definitions, first-hand source verification of every estimate, pre-registration, multi-paradigm triangulation, and formal information-size and certainty assessment. Limitations include high statistical heterogeneity; residual confounding inherent to observational cohorts (one cohort adjusted for age and sex only); reliance on study-level rather than individual-patient data, precluding adjustment for iron status or anaemia severity beyond the binary threshold; one ICD-9-coded anaemia definition; and the inability, with a study-level synthesis, to test whether anaemia carries differential prognostic weight across the recognized HFpEF phenogroups (obese-cardiometabolic, cardiorenal-elderly, right-heart-pulmonary-vascular^2^), a question for individual-patient-data meta-analysis or registry stratification. The two externally anchored Bayesian priors (Majmundar 2021, Berry MAGGIC) are not independent of our likelihood; the Majmundar synthesis pooled nine of our cohorts plus the Berry estimate, so those posteriors are reported only as robustness-to-anchoring checks, not as independent corroboration, and the weakly informative posterior is the reference. Certainty is moderate, not high.

## Conclusions

WHO-defined anaemia is independently associated with a ∼40%–50% higher all-cause mortality in HFpEF, and this association is preserved under the contemporary LVEF ≥50% definition. The evidence base is informationally adequate and of moderate certainty. Anaemia warrants recognition as a prognostic marker in HFpEF, and the mortality signal supports mortality-powered interventional trials.

## Supplementary Material

xvag213_Supplementary_Data

## Declarations

### Data Availability

All data underlying this meta-analysis were extracted from previously published studies and are presented in full within the article and its Supplementary material.

### Pre-registered Clinical Trial Number

None supplied.
