# Supplementary material for: Anaemia and all-cause mortality in contemporary heart failure with preserved ejection fraction (left ventricular ejection fraction ≥50%): an updated systematic review and meta-analysis
Source: ESC Heart Fail. 2026 Sep 8;13(5):xvag213. doi: 10.1093/eschf/xvag213 (PMC13650565; doi:10.1093/eschf/xvag213)
Supplement: Supplementary file 1 [file xvag213_supplementary_data.zip › ESCHF-26-00695R1_Supplementary_Material.docx]

Supplementary material for: Anaemia and All-Cause Mortality in Contemporary HFpEF (LVEF ≥50%): An Updated Systematic Review and Meta-Analysis. Tripathi S, Jain D. ESC Heart Failure. Manuscript ESCHF-26-00695R1.

# SUPPLEMENTARY MATERIALS

## PRISMA 2020 Checklist

| # | Item | Location |
| --- | --- | --- |
| 1 | Title — identifies as systematic review | Title |
| 2 | Abstract — structured summary | Abstract |
| 3 | Rationale | Introduction |
| 4 | Objectives (PICO/PECO) | Introduction (final paragraph); §S1.3 |
| 5 | Eligibility criteria | Methods §2; §S1.3 |
| 6 | Information sources | Methods §2; §S1.2 |
| 7 | Search strategy (full) | §S1.2 |
| 8 | Selection process | Methods §2; §S1.4 |
| 9 | Data collection process | §S1.4 |
| 10 | Data items | §S1.4; Table 1 |
| 11 | Risk-of-bias assessment (ROBINS-E) | Methods §2; §3.2; Supp Fig S1 |
| 12 | Effect measures (aHR) | Methods §2; §S1.3 |
| 13 | Synthesis methods (PM + HKSJ) | Methods §2; §S1.5 |
| 14 | Reporting-bias assessment | §3.6; §S1.5; Supp Tab S7-S9 |
| 15 | Certainty assessment (GRADE) | §3.9; §S1.6; Table 3 |
| 16 | Study selection results | §3.1; Figure 1 |
| 17 | Study characteristics | §3.2; Table 1 |
| 18 | Risk of bias in studies | §3.2; Supp Fig S1 |
| 19 | Results of individual studies | Table 1; Figure 2 |
| 20 | Results of syntheses | §3.3-§3.8; Figures 2-3 |
| 21 | Reporting biases | §3.6; Supp Tab S7-S9; Supp Fig S5-S6 |
| 22 | Certainty of evidence | §3.9; Table 3; Supp Tab S14 |
| 23 | Discussion | §4 |
| 24 | Registration and protocol | PROSPERO CRD420251361160 |
| 25 | Support/funding | Funding |
| 26 | Competing interests | Conflicts of interest |
| 27 | Data/code availability | Data availability |

## Supplementary Methods (full)

### §S1.1 PRISMA 2020 compliance

The review followed the PRISMA 2020 statement; the completed 27-item checklist is above. The protocol was prospectively registered (PROSPERO CRD420251361160) before data extraction.

### §S1.2 Search strategy

Five databases (PubMed/MEDLINE, Embase [Ovid and Elsevier], Web of Science Core Collection, Scopus, and Cochrane CENTRAL) were searched from inception to 2026, combining controlled vocabulary and free-text terms for HFpEF, anaemia/haemoglobin, and mortality/prognosis. Scopus was added beyond the four databases named in the registered protocol; this addition is logged here as a deviation (§S1.5.0) and broadened, rather than restricted, retrieval. Reference lists of included studies and prior reviews were hand-searched.

### §S1.3 Eligibility — effect-measure requirement

A study was eligible for the quantitative synthesis only if it reported an adjusted hazard ratio (or an estimate validly convertible to one) for binary WHO-defined anaemia (Hb <13 g/dL men, <12 g/dL women) versus non-anaemic patients, with all-cause mortality as the outcome, in a HFpEF stratum at the study-defined ejection-fraction threshold. The following were explicitly ineligible: (i) logistic-regression odds ratios that cannot be converted to a time-to-event hazard ratio (for a common outcome the rare-outcome OR≈RR≈HR approximation fails); (ii) effect estimates from non-Cox models that do not yield an interpretable hazard ratio; (iii) mixed-ejection-fraction cohorts without an HFpEF-stratified estimate; (iv) combined anaemia-and-iron-deficiency exposure strata; and (v) estimates available only at second hand (e.g., read from a prior meta-analysis's forest plot) or without a locatable primary source.

### §S1.4 Selection and extraction (primary-source standard)

Records were screened in duplicate in Rayyan (S.T., D.J.); Cohen's κ was 0.56 pre-adjudication and 1.00 after consensus. Every candidate study's effect estimate was verified against its own full text before pooling, confirming the regression model (Cox vs logistic/other), the effect measure as reported, the exact adjusted point estimate and 95% CI (and, where relevant, the p-value), the binary WHO anaemia exposure, the all-cause-mortality outcome, and the HFpEF stratum at the stated EF threshold. Estimates reported only in a prior meta-analysis were not accepted. First-hand verification corrected several extraction values: Grigorian 2005 point/CI taken directly from the source table (2.647, 1.308-5.357); Lund 2014 upper CI corrected to 2.62; Caughey 2014 analytic N corrected to 6,414; Gupta 2020 confirmed 1.40 (1.14-1.74) from the results table; Savarese 2020 confirmed 1.28 (1.20-1.36) (an earlier 1.31 [1.19-1.45] was a cross-cell transcription error); Streng 2018 estimate available only from the published forest plot and flagged accordingly.

### §S1.5 Statistical analysis

Co-primary pools A (LVEF ≥40%) and B (LVEF ≥50%) were synthesised on the log-hazard-ratio scale by random-effects models with the Paule-Mandel τ² estimator and Hartung-Knapp-Sidik-Jonkman variance correction (R 4.6.0, *metafor*). We report the pooled aHR, 95% CI, 95% prediction interval, I², and τ. Bayesian random-effects analysis used *bayesmeta* under four pre-specified priors on the pooled log-hazard-ratio, all sharing a half-Normal(0.5) prior on between-study heterogeneity τ: (i) weakly-informative N(0, 10²); (ii) a Majmundar 2021-anchored N(0.365, 0.17²) = N(log 1.44, 0.17²); (iii) a Berry MAGGIC IPD-anchored N(0.513, 0.17²) = N(log 1.67, 0.17²); and (iv) a sceptical N(0, 0.35²). We report the posterior median, 95% credible interval, and P(HR>1). The two externally-anchored priors are not independent of the likelihood — the Majmundar 2021 synthesis pooled nine of our Pool-A cohorts plus the Berry MAGGIC estimate, and MAGGIC is an individual-patient-data synthesis that may share cohorts with ours — so these analyses are interpreted as robustness-to-anchoring checks, with the weakly-informative prior as the reference; both anchors (1.44, 1.67) lie at or above the data and can therefore only pull the posterior upward. A robust Bayesian model-average (*RoBMA*) combined 36 component models spanning effect-present/absent, heterogeneity-present/absent, and publication-bias-present/absent, on the log-ratio scale. Small-study effects were assessed with Egger/FAT-PET and PEESE-RE regression, a Vevea-Hedges step-function selection model, the Ioannidis-Trikalinos test for excess significance, cumulative meta-analysis, and trim-and-fill. A secondary per-1 g/dL-lower-Hb dose-response pool (k = 6) was synthesised by the same RE method. The diversity-adjusted required information size was computed across baseline-mortality (20/30/40%) and HR-anchor (1.20/1.30/1.49) scenarios, with diversity D². Certainty was rated with the GRADE prognostic-factor framework (Foroutan 2020), starting at high for cohort evidence.

**§S1.5.0 Deviation log.** (1) Scopus added beyond the four registered databases (broadened retrieval). (2) Dual co-primary EF definitions (≥40% and ≥50%) formalised to address the contemporary guideline threshold. (3) RoBMA effect sizes entered on the log-ratio (OR) scale as a pragmatic approximation for the log-HR, standard for ratio measures in this implementation.

### §S1.6 GRADE for prognostic factors

Per Foroutan 2020, cohort/registry evidence for a prognostic factor begins at high certainty. We downgraded one level for risk of bias (eleven of seventeen cohorts moderate on ROBINS-E, with variable adjustment, one cohort adjusted for age and sex only, one ICD-coded anaemia definition, one graphically-extracted estimate, and two conference-abstract sources; none high or critical) and one level for inconsistency (I² ≈ 85% with unexplained residual heterogeneity). We judged indirectness and imprecision not serious; small-study effects were detected but the bias-corrected estimates were stable, so publication bias was carried as a limitation rather than a separate downgrade. We applied a one-level upgrade for the graded per-1 g/dL-lower-haemoglobin dose-response relationship. Final certainty: Moderate; a more conservative assessor who declines the dose-response upgrade would rate it Low.

### §S1.7 Cohort-identity audit

Two Veterans Affairs/registry cohorts (Veterans-HFpEF 2017 and Ather 2012) were screened for participant overlap; SA-8 removes Veterans-HFpEF from Pool B (k = 11) as the conservative bound. Three Swedish HF Registry analyses (Savarese 2020, Ugarph-Morawski 2025, Lund 2014) draw on overlapping registries over different periods/strata and were retained as distinct analytic estimates.

### §S1.8 Study excluded at full-text verification

**Köseoğlu and Özlek 2024 (*Diagnostics* 2024;14:209) — excluded.** Although labelled a "hazard ratio" (5.40, 95% CI 4.30-6.21), the multivariable analysis was a logistic-regression model, so the estimate is an adjusted odds ratio. With 28% all-cause mortality (54% among anaemic patients), the outcome is common and the odds ratio overstates and is not convertible to a hazard ratio; the crude OR reconstructed from the published 2×2 data (8.55) matches the reported univariable value, confirming the measure. The reported CI is internally inconsistent with the stated p = 0.001 (the interval implies SE ≈ 0.09, whereas p = 0.001 implies SE ≈ 0.51, i.e., ~1.98-14.75), and the study carried a High overall ROBINS-E rating. Exclusion was on the pre-specified effect-measure ground, not on the magnitude of the estimate or its statistical influence.

**§S1.8.1 Initially flagged but verified and retained.** *Iorio 2018:* the value (1.90, 1.40-2.60) had been taken from a prior review's forest plot; the primary report confirmed the HFpEF-stratum (LVEF ≥50%, n = 1,373) Cox aHR (Table 3), adjusted for age and sex only (noted in ROBINS-E). *Pintér 2022* (previously mis-cited "Csikós"): aHR 2.33 (1.21-4.52), adjusted age/sex/creatinine/furosemide, LVEF ≥40% cohort (n = 375; 87% HFpEF), no ≥50%-only estimate → Pool A only. *Chairat 2020:* a completeness gap (present among screening includes, absent from the prior analysis set); verified HFpEF (LVEF ≥50%, n = 287) Cox aHR 2.667 (1.216-5.853) for 1-year all-cause mortality, adjusted age/sex/smoking/ACEI-ARB/β-blocker/hypertension/Charlson. After the single exclusion, 17 studies entered Pool A and 12 entered Pool B.

## Supplementary Tables

| S-Table | Title |
| --- | --- |
| S1 | Studies excluded at full text, with reasons (Köseoğlu) |
| S2 | Per-study random-effects weights, Pools A & B |
| S3 | Full sensitivity-analysis numeric detail |
| S4 | Leave-one-out diagnostic, both pools |
| S5 | Bayesian RE, 4 priors × 2 pools (median, 95% CrI, P(HR>1), τ) |
| S6 | RoBMA model-averaged estimates (+ S6b inclusion components) |
| S7 | Vevea-Hedges selection model |
| S8 | PEESE-RE / FAT-PET regression |
| S9 | Test for excess significance |
| S10 | Per-1 g/dL dose-response pool (k = 6) |
| S11 | Univariable meta-regression (all moderators) |
| S12 | EF-permissiveness descriptive contrast (<50 vs ≥50) |
| S13 | DIS sensitivity (baseline rate × HR anchor) |
| S14 | Summary of Findings (Foroutan 2020 prognostic SoF) |

### Supplementary Table S1 — Study excluded at full-text verification

| Study | EF cut | N | Reported estimate | Reason for exclusion |
| --- | --- | --- | --- | --- |
| Köseoğlu 2024 | ≥50 | 212 | OR 5.40 (4.30-6.21) | Logistic-regression adjusted **odds ratio** mislabelled "HR" for a common (28%) outcome → not convertible to a hazard ratio; protocol-ineligible (PROSPERO §S1.3). Also High ROBINS-E; reported CI incompatible with stated p = 0.001. |

### Supplementary Table S2 — Per-study random-effects weights

| Study | Pool A weight % | Pool B weight % |
| --- | --- | --- |
| Grigorian 2005 | 1.55 | 1.65 |
| Felker 2006 | 8.33 | — |
| Tehrani 2009 | 4.16 | 4.87 |
| Ather 2012 | 7.73 | 10.48 |
| Caughey 2014 | 5.87 | — |
| Lund 2014 | 4.51 | — |
| PEOPLE 2016 | 3.14 | 3.54 |
| Veterans-HFpEF 2017 | 10.41 | 16.04 |
| Iorio 2018 | 4.99 | 6.02 |
| Streng 2018 | 4.58 | 5.44 |
| Jin 2019 | 5.32 | 6.51 |
| Chairat 2020 | 1.29 | 1.36 |
| Gupta 2020 | 6.94 | — |
| Savarese 2020 | 10.08 | 15.28 |
| Pintér 2022 | 1.74 | — |
| Qiu 2023 | 10.04 | 15.18 |
| Ugarph-Morawski 2025 | 9.32 | 13.62 |

No single study exceeds ~16% weight; weights are also encoded in the Figure 2 forest-plot square sizes.

### Supplementary Table S3 — Full sensitivity-analysis numeric detail (SA-1…SA-12)

Reproduces Table 2 with the pre-specified analysis descriptions; see Table 2 for values. SA-4 is the outlier/influence diagnostic (no outlier detected: max studentized residual |z| = 1.99 < Bonferroni threshold 2.97); SA-5 (outlier-excluded re-pool) is conditional on SA-4 and was therefore not performed; SA-9-SA-12 are the publication-bias suite (S-Tables 6-9).

### Supplementary Table S4 — Leave-one-out diagnostic (both pools)

| Omitted study | Pool A HR (95% CI) | I² | Pool B HR (95% CI) | I² |
| --- | --- | --- | --- | --- |
| Grigorian 2005 | 1.48 (1.34-1.63) | 84% | 1.39 (1.27-1.52) | 75% |
| Felker 2006 | 1.49 (1.33-1.67) | 87% | — | — |
| Tehrani 2009 | 1.50 (1.34-1.67) | 88% | 1.40 (1.24-1.58) | 87% |
| Ather 2012 | 1.51 (1.35-1.69) | 87% | 1.42 (1.25-1.61) | 87% |
| Caughey 2014 | 1.45 (1.32-1.59) | 80% | — | — |
| Lund 2014 | 1.48 (1.33-1.65) | 86% | — | — |
| PEOPLE 2016 | 1.50 (1.35-1.68) | 87% | 1.42 (1.25-1.60) | 88% |
| Veterans-HFpEF 2017 | 1.50 (1.34-1.68) | 79% | 1.39 (1.23-1.58) | 76% |
| Iorio 2018 | 1.47 (1.33-1.64) | 85% | 1.38 (1.26-1.51) | 75% |
| Streng 2018 | 1.51 (1.36-1.68) | 86% | 1.42 (1.27-1.60) | 85% |
| Jin 2019 | 1.51 (1.37-1.68) | 85% | 1.43 (1.28-1.59) | 83% |
| Chairat 2020 | 1.48 (1.34-1.64) | 85% | 1.39 (1.27-1.53) | 78% |
| Gupta 2020 | 1.51 (1.35-1.69) | 87% | — | — |
| Savarese 2020 | 1.52 (1.36-1.69) | 81% | 1.43 (1.27-1.62) | 80% |
| Pintér 2022 | 1.48 (1.34-1.64) | 85% | — | — |
| Qiu 2023 | 1.52 (1.36-1.69) | 82% | 1.43 (1.26-1.62) | 81% |
| Ugarph-Morawski 2025 | 1.51 (1.35-1.69) | 86% | 1.41 (1.24-1.61) | 86% |
| All five reviewer-flagged removed (k = 12) | 1.54 (1.33-1.78) | 78% | 1.42 (1.15-1.75) [k = 7] | 81% |

Pool A range 1.45-1.52; Pool B range 1.38-1.43 — no single study is influential enough to overturn the result.

### Supplementary Table S5 — Bayesian random-effects (4 pre-specified priors × 2 pools)

| Pool | Prior | Posterior median HR | 95% CrI | τ (median) | P(HR>1) |
| --- | --- | --- | --- | --- | --- |
| A | Weakly-informative N(0, 10²) | 1.48 | 1.35-1.65 | 0.130 | 1.000 |
| A | Majmundar-anchored N(0.365, 0.17²) | 1.48 | 1.36-1.63 | 0.128 | 1.000 |
| A | Berry MAGGIC-anchored N(0.513, 0.17²) | 1.49 | 1.37-1.66 | 0.133 | 1.000 |
| A | Sceptical N(0, 0.35²) | 1.47 | 1.35-1.63 | 0.128 | 1.000 |
| B | Weakly-informative N(0, 10²) | 1.40 | 1.28-1.56 | 0.098 | 1.000 |
| B | Majmundar-anchored N(0.365, 0.17²) | 1.40 | 1.29-1.55 | 0.097 | 1.000 |
| B | Berry MAGGIC-anchored N(0.513, 0.17²) | 1.42 | 1.30-1.58 | 0.100 | 1.000 |
| B | Sceptical N(0, 0.35²) | 1.39 | 1.27-1.54 | 0.097 | 1.000 |

The Majmundar/Berry-anchored priors are not independent of the likelihood (overlap; §S1.5) and are interpreted as robustness-to-anchoring, with the weakly-informative prior as reference.

### Supplementary Table S6 — Robust Bayesian Meta-Analysis (model-averaged, Pool A)

| Parameter | Mean | 95% CrI (log) | HR scale |
| --- | --- | --- | --- |
| μ (pooled effect) | 0.334 | 0.154-0.460 | **HR 1.41 (1.17-1.58)** |
| τ (heterogeneity) | 0.133 | 0.053-0.288 | — |

**Inclusion Bayes factors (S6b):** effect present **BF = 79.0** (post-prob 0.988); heterogeneity present **BF = 3999** (1.000); publication bias present **BF = 4.28** (0.811). The effect survives model-averaging across null-effect and publication-bias models.

### Supplementary Table S7 — Vevea-Hedges 4-parameter selection model

| Parameter | Estimate |
| --- | --- |
| Adjusted intercept (log HR) | 0.352 |
| **Adjusted HR** | **1.42** |
| τ² | 0.011 |
| Selection weight (p < 0.025) | 1.000 |
| Selection weight (p ≥ 0.025) | 0.249 |

### Supplementary Table S8 — PEESE-RE / FAT-PET regression

| Model | Estimate | p-value |
| --- | --- | --- |
| FAT-PET (Egger): slope on SE | 1.23 | 0.031 |
| FAT-PET adjusted HR (intercept) | 1.32 | <0.001 |
| PEESE: slope on variance | 4.08 | 0.019 |
| **PEESE-RE adjusted HR (intercept)** | **1.39** | <0.001 |

### Supplementary Table S9 — Test for Excess Significance

| Metric | Value |
| --- | --- |
| Observed significant (O) | 14 |
| Expected significant (E) | 12.19 |
| O/E ratio | 1.15 |
| z (Poisson-binomial approx) | 1.29 |
| p-value | 0.20 (negative — no excess) |

### Supplementary Table S10 — Per-1 g/dL lower-haemoglobin dose-response pool (k = 6)

| Study | HR per 1 g/dL (95% CI) | N |
| --- | --- | --- |
| Okuno 2019 | 1.25 (1.02-1.52) | 535 |
| Tada 2008 | 1.33 (1.19-1.47) | 357 |
| Young 2008 | 1.08 (1.03-1.13) | 48,612 |
| Caughey 2014 | 1.20 (1.10-1.30) | 6,339 |
| Felker 2006 | 1.17 (1.06-1.29) | 3,093 |
| Gupta 2020 | 1.13 (1.05-1.22) | 1,748 |
| **POOLED (k = 6)** | **1.17 (1.08-1.27)** | 60,684 |

### Supplementary Table S11 — Univariable meta-regression (all moderators, Pool A)

| Moderator | β | 95% CI | p | R² |
| --- | --- | --- | --- | --- |
| EF threshold (≥50 vs <50) | −0.196 | −0.399 to 0.007 | 0.057 | 36.0% |
| Publication year | −0.013 | −0.031 to 0.005 | 0.135 | 18.8% |
| Follow-up (months) | −0.002 | −0.009 to 0.004 | 0.505 | 0% |
| Mean age | −0.002 | −0.025 to 0.022 | 0.869 | 0% |
| Anaemia prevalence (%) | 0.005 | −0.005 to 0.014 | 0.329 | 0.3% |
| % male | −0.002 | −0.008 to 0.003 | 0.405 | 0% |

### Supplementary Table S12 — EF-permissiveness descriptive contrast

| Stratum | k | HR (95% CI) | 95% PI | I² | Q (p) |
| --- | --- | --- | --- | --- | --- |
| Permissive <50% | 5 | 1.71 (1.37-2.14) | 1.15-2.54 | 45% | 7.2 (0.13) |
| Strict ≥50% | 12 | 1.41 (1.26-1.57) | 1.06-1.87 | 83% | 46.2 (<0.001) |

### Supplementary Table S13 — DIS (diversity-adjusted required information size)

Accrued N = 109,909; I² = 85.3%; D² = 0.85. AIS = single-trial information size; DIS = diversity-adjusted.

| Baseline mortality | HR anchor | AIS | DIS |
| --- | --- | --- | --- |
| 20% | 1.49 | 1,117 | 7,605 |
| 30% | 1.49 | 761 | 5,181 |
| 40% | 1.49 | 589 | 4,010 |
| 20% | 1.30 | 2,735 | 18,620 |
| 30% | 1.30 | 1,853 | 12,615 |
| 40% | 1.30 | 1,423 | 9,688 |
| 20% | 1.20 | 5,862 | 39,907 |
| 30% | 1.20 | 3,960 | 26,959 |
| 40% | 1.20 | 3,029 | 20,621 |

The accrued sample (109,909) exceeds the largest DIS scenario (39,907) ≈ 2.8-fold; the evidence base is informationally adequate.

### Supplementary Table S14 — Summary of Findings (Foroutan 2020 prognostic-factor SoF)

| Outcome | Participants (studies) | Pooled aHR (95% CI) | 95% PI | Certainty |
| --- | --- | --- | --- | --- |
| All-cause mortality | 109,909 (k = 17), Pool A | 1.49 (1.35-1.66) | 1.07-2.09 | Moderate |

## Supplementary Figures

All supplementary figures are also provided as png (300 dpi), svg (vector), jpg, and — where applicable — interactive html in output/figures/.

**Supplementary Figure S1.** ROBINS-E risk-of-bias heat-map (D1-D7 × k = 17).

**Alt text:** Risk-of-bias heat-map across seven ROBINS-E domains for 17 studies, predominantly low to moderate concern and none high.

**Supplementary Figure S2.** Leave-one-out forest (Pool A).

**Alt text:** Leave-one-out forest plot for Pool A; the pooled estimate stays between 1.45 and 1.52 whichever single study is removed.

**Supplementary Figure S3.** Cumulative meta-analysis forest (Pool A, by year).

**Alt text:** Cumulative meta-analysis forest by year; the pooled estimate stabilises near 1.5 from 2014 onward.

**Supplementary Figure S4.** RoBMA model-averaged posterior of μ (with prior overlay).

**Alt text:** Robust Bayesian model-averaged posterior of the pooled effect with prior overlay; model-averaged aHR 1.41.

**Supplementary Figure S5.** Funnel plot (Pool A).

**Alt text:** Funnel plot for Pool A showing mild asymmetry consistent with modest small-study effects.

**Supplementary Figure S6.** PEESE-RE small-study-effect regression.

**Alt text:** PEESE small-study-effect regression; bias-corrected aHR 1.39.

**Supplementary Figure S7.** Per-1 g/dL dose-response forest (k = 6).

**Alt text:** Per-1 g/dL lower-haemoglobin dose-response forest of six cohorts; pooled aHR 1.17.

**Supplementary Figure S8.** Meta-regression bubble plots (EF threshold + covariates).

**Alt text:** Meta-regression bubble plots for the ejection-fraction threshold and other study-level covariates.
